# A cost-effective handheld breast scanner for use in low-resource environments: a validation study

**DOI:** 10.1186/s12957-016-1022-2

**Published:** 2016-10-28

**Authors:** Robyn B. Broach, Rula Geha, Brian S. Englander, Lucy DeLaCruz, Holly Thrash, Ari D. Brooks

**Affiliations:** 1 Department of Surgery, University of Pennsylvania School of Medicine, 3400 Spruce Street, 4 Silverstein, Philadelphia, PA 19104 USA; 2 Department of Radiology, Pennsylvania Hospital, 800 Spruce Street, Philadelphia, PA 19106 USA; 3 UE Lifesciences Inc., 3711 Market St, Suite 800, Philadelphia, PA 19104 USA; 4 Department of Surgery, University of Pennsylvania School of Medicine, 800 Walnut Street, 20th Floor, Philadelphia, PA 19107 USA

## Abstract

**Background:**

With the incidence of breast cancer rising worldwide, we are evaluating the iBreastExam (iBE) (UE LifeSciences Inc.), a handheld breast scanning device that can be utilized by community health workers to screen for breast abnormalities. The purpose of this study is to determine the sensitivity of the iBE in a population undergoing diagnostic breast imaging.

**Methods:**

Adult patients presenting to a breast imaging center for a diagnostic workup were eligible. Patients underwent an iBE exam performed by a trained ultrasound technician followed by their indicated imaging. Demographic, imaging, and biopsy data were recorded.

**Results:**

Seventy-eight iBE exams were completed, 77 females and one male with a mean age of 42 (21–79). All patients were evaluated by ultrasound, 52 had diagnostic mammography and 39 had biopsies. Imaging and/or biopsy confirmed a mass (fibroadenoma, cyst, papilloma, myofibroblastoma, fat necrosis, DCIS, or cancer) in 60 patients. Twelve patients had a cancer diagnosed. In total, 342 quadrants were scanned, 77 quadrants had lesions confirmed on imaging, and iBE correctly identified 66 lesions for a sensitivity of 86 % and specificity of 89 %.

**Conclusions:**

This validation study demonstrated excellent sensitivity of iBE for the identification of clinically significant lesions in patients presenting for diagnostic imaging.

**Trial registration:**

A Cost-Effective Handheld Breast Scanner for Use in Low Resource Environments: A Validation Study: NCT02814292.

## Background

The incidence of breast cancer is rising rapidly worldwide. Since 2008, the incidence of breast cancer has increased by more than 20 % worldwide [1]. Globally, breast cancer now represents one in four of all cancers among women. Early detection improves the survival rate, makes treatment less costly, and lowers the overall burden of the disease. The iBreastExam (iBE) device was developed as a pre-screening tool that could identify women in need of further breast imaging without requiring extensive breast screening infrastructure. This inexpensive handheld device uses piezoelectric palpation to enhance the clinical breast exam (CBE) for detection of breast masses that require further investigation.

The iBE device was built on the principle of the piezoelectric finger (PEF) detector. After their initial development, the PEF was proven in bench-top work on breast phantoms and subsequently in excised human tumors with excellent detection ability and size prediction [2]. A pilot in vivo clinical trial was then completed using a very basic array of four PEFs with excellent detection of breast lesions in women undergoing clinical evaluation [3]. Subsequently, the iBE device was developed as a 16 finger array with a rapid wireless mobile processor algorithm and durable battery powered handpiece. The device was developed to be operated by a technician or health care worker and does not require a radiologist for interpretation. This prospective study was designed specifically to validate the ability of the iBE device to detect breast abnormalities worthy of further diagnostic imaging.

## Methods

### Study design

Approval was obtained from the University of Pennsylvania Institutional Review Board (IRB, protocol # 819121) and the Abramson Cancer Center Clinical Trials Scientific Review Committee (CTSRMC, UPCC # 33113) for this study. All patients signed informed consent prior to participation.

Eighty-nine patients with suspected breast lesions were consented and examined with the iBE device from August 2014 through January 2015. Patients participated for one clinic visit during which the iBE device was used to examine the areas of concern as well as uninvolved areas of the breast. Eleven patients were excluded from the analysis due to missing the iBE data. Missing iBE data was a result of incomplete data transfer from the iBE. If the wireless communication of the device was incomplete, repeat scans were not attempted due to time constraints of the high volume at the breast imaging center. Results from the iBE did not influence or change the medical care given to the patients.

Women and men 18 years of age or older with symptomatic breast masses or women with asymptomatic breast masses discovered by palpation or imaging were included. Patients under 18 years of age were excluded.

The current pathway of breast lesion screening and detection at this institution begins with a screening mammogram in asymptomatic women. In women with a negative screening mammogram, the pathway is complete and a regular interval follow-up is recommended. This tends to be the case even in women with dense breast tissue. In women with an abnormal screening mammogram or palpable masses from clinical breast exam, a diagnostic mammogram is done in women over 40 and an ultrasound in all women. Typically, if a woman is under 35 with a palpable mass, mammography is skipped and ultrasound is the diagnostic test of choice. If the additional imaging reveals something suspicious, a biopsy may be recommended for confirmation. Since the inclusion criteria required the participating patients to have a mass detected by palpation or imaging, the majority of the patients were recommended for further imaging. All follow-up diagnostic information was extracted from the electronic medical record and recorded. The type and number of diagnostic techniques were determined by the radiologist and/or the surgical oncologist based on the results of the previous exams, not including the iBE.

#### Classification

Initially in this study, a positive or a negative finding was defined only by the Breast Imaging-Reporting and Data System (BIRADS) classification assigned by the radiologist interpreting the results of the diagnostic technique(s). BIRADS 1 and 2 defined a negative detection while BIRADS 0, 3, 4, 5, and 6 defined a positive detection or clinically relevant finding. However, it was discovered that many of the radiology reports classified as BIRADS 1 or 2 reported a measurable finding such as a cyst. Since the iBE was designed to be a pre-screening tool with the intent to identify all lesions, not just cancerous lesions, the positive/negative classification for this study was revised to include the presence or absence of clinically relevant detectable lesions as follows: negative findings included negative study, fibrocystic changes, benign calcification, and gynecomastia while positive findings included fibroadenoma, cyst, myofibroblastoma, fat necrosis, papilloma, ductal carcinoma in situ (DCIS), or cancer. Thus, when analyzing the iBE results by independent quadrants, each iBE quadrant was compared to the correlating quadrant of a mammography, ultrasound, biopsy, or MRI. A positive finding was defined as the presence of a measurable finding in that quadrant independent of BIRADS classification.

### Device

**Fig. 1:**
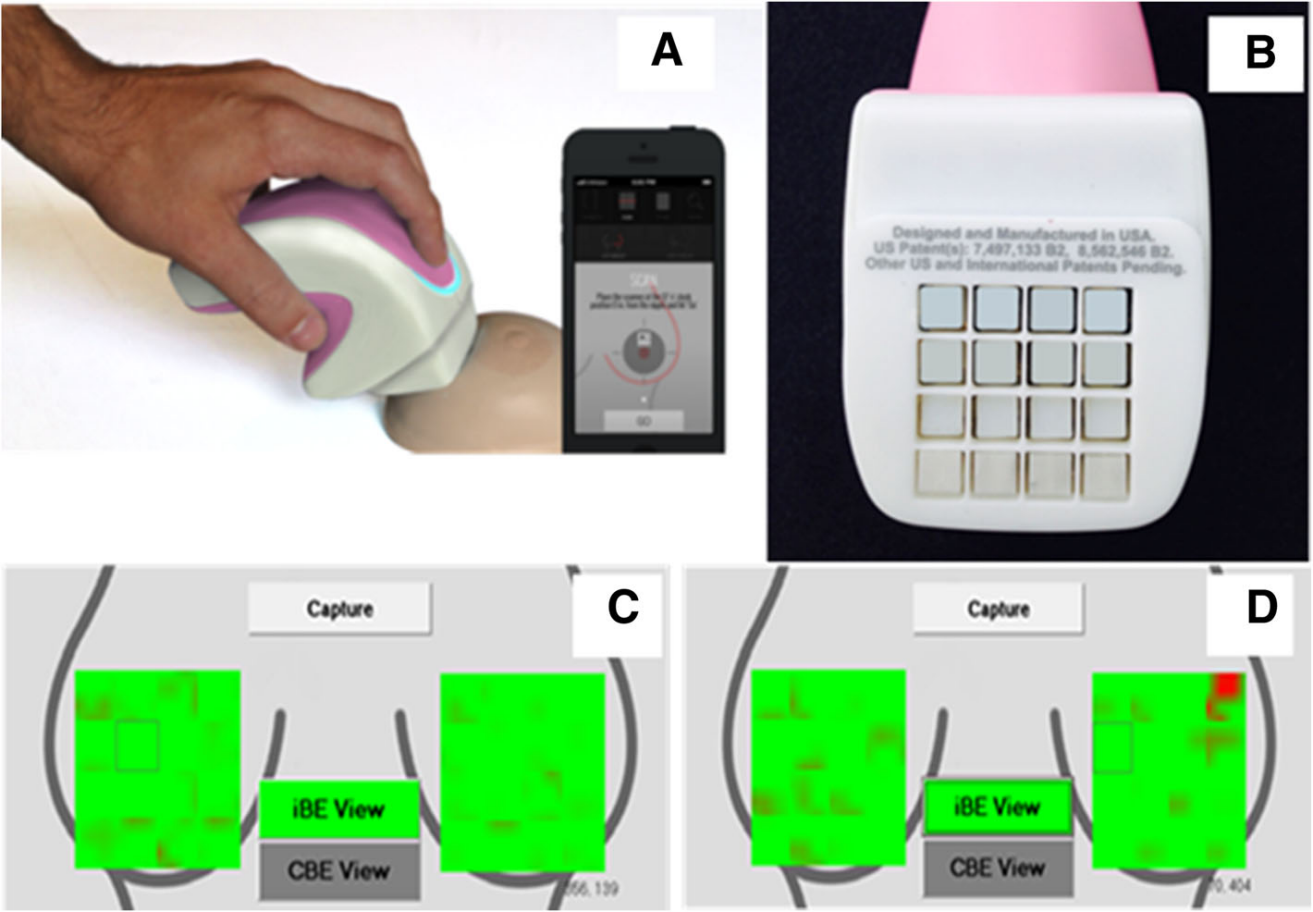
**a** iBE device and the device in use. **b** The 4 × 4 array of PEFS. **c** Data pressure map illustration of normal or negative results. **d** Data pressure map illustration of abnormal or positive results

The iBreastExam™ or iBE™ (Fig. 1a, b) consists of a handheld compression probe containing a 4 × 4 array of piezoelectric tactile pressure sensors, a custom built electronic board and a tablet. The iBE communicates wirelessly with a mobile device to display and store the findings in real time. Compression data are recorded as a unique file at the end of every scan to the mobile device which is synced to an encrypted database via Dropbox where a copy is saved. No identifiable data are collected or stored by the iBE software.

The iBE evaluations were performed by an ultrasound technologist who was trained to use the iBE by the manufacturer and the principal investigator of the study. Training involved 1 h of education about the functions of the device, practice on a breast phantom, and then 10 observed iBE studies with the principal investigator. The results of the iBE were displayed on the touch screen in a pressure map fashion (Fig. 1c, d). Green indicates normal breast tissue while red indicates a lesion was detected meaning the iBE is suggesting further testing to characterize the lesion, as the iBE does not differentiate the type of lesion it detects. The patient lies in the supine position while the test was done. The device was calibrated on the breast in an uninvolved area; it can be recalibrated multiple times. The iBE records the data and collects into 4 × 4 array map of the breast, and each individual square represents the 4 × 4 array of the PEFS. This map was divided into sectors demarcated by three consecutive hours of a clock in order to directly compare to the clock positioning assigned by mammography or ultrasonography.

## Results

The mean age of the patients was 42 years old (age range 21 to 79 years). The racial distribution of the patient population was 51 % Caucasian, 35 % African American, 3 % Asian, 5 % Hispanic, and 6 % Unknown. One male participated in the study. All 78 patients underwent ultrasonography while only 52 underwent mammography and 39 underwent biopsies (50 %).

The iBE results were evaluated by definitive quadrants. The definitive quadrants break down the superimposed clock into four equal sectors (e.g., 12–3, 3–6, 6–9, 9–12). The quadrants were defined when the iBE identified a positive finding; the positive finding became the center of a quadrant, meaning if a positive finding was identified at 5:00, the first quadrant would be 3:30–6:30 and the remaining quadrants defined as follows 6:30–9:30, 9:30–12:30, and 12:30–3:30.

Through imaging, 77 of the quadrants were determined as a positive detection and 265 as negative findings with an overall total of 342 quadrants evaluated by the iBE (Table 1). Out of the 77 positive quadrants, 66 were identified correctly (true positive) by the iBE resulting in a Sn = 85.7 %, and 237 out of 265 quadrants were correctly characterized normal (true negative) resulting in Sp = 89.4 %. Also, illustrated in Table 1 are the positive detection rate, negative detection rate, and accuracy, positive predictive value (PPV) = 70.2 %, negative predictive value (NPV) = 95.6 %, and accuracy (Acc) = 88.6 %, respectively.

**Table 1:** iBE accuracy measures by definitive quadrants

Total definitive quadrants *N* = 342	Mass positive	Mass negative	Measures
Test positive quadrant	66	28	PPV = 70.2 %
Test negative quadrant	11	237	NPV = 95.6 %
Measures	Sn = 85.7 %	Sp = 89.4 %	Accuracy = 88.6 %

Table 2 demonstrates the distribution of BIRADS categories according to the patient’s final report of the evaluation of the entire breast. BIRADS (1 and 2) were assigned to 30 patients, and BIRADS (3, 4, 5, and 6) were assigned to 48 patients. A total of 5 masses went undetected by the iBE, all of which were under 1 cm in size. Of the false negatives, 3 false negatives were classified as BIRADS 3, noncancerous findings while the remaining 2 were cancerous and classified as BIRADS 4 until biopsies were completed. Two of the 3 noncancerous false negatives were fibroadenomas, and the remaining 1 was stromal calcification. One cancerous false negative was retroareolar 0.5-cm ductal carcinoma in situ, and the second was 0.7-cm mucinous carcinoma located at 8:30, 6 cm from the nipple.

**Table 2:** Distribution of BIRADS and number of FN in each category

BIRADS	Final report	No. of FN
1	3 (4 %)	0
2	27 (35 %)	0
3	26 (33 %)	3
4	13 (16.7 %)	2
5	1 (1.3 %)	0
6	8 (10 %)	0
Total	78	5

### Malignancy

Of the 78 patients, 12 patients were found to have a malignancy. Ten of the 12 cancerous diagnoses were detected by the iBE (Sn = 83 %, Sp = 74.5 %) while 2 were undetected (Table 3). The size of malignancies detected ranged from of 0.9 to 3.8 cm with a mean size of 1.91 cm ± 0.9. The two undetected by the iBE mean size was 0.6 ± 0.1 cm. One of the undetected malignancies was ductal carcinoma in situ; the other was mucinous carcinoma. Out of the 10 malignant masses detected by the iBE, 6 were invasive ductal carcinoma, 2 were invasive mammary carcinoma and 1 was ductal carcinoma in situ. Two of the detected masses were located in the retroareolar region.

**Table 3:** iBE accuracy for malignancy detection

	No cancer	Cancer	Total
IBE (+)	84	10	94
IBE (−)	246	2	248
Total	330	12	342
Measures	Sn = 83 %	Sp = 74.5 %	

Diagnostic mammograms were performed on 11 of the 12 cancers diagnosed, while one patient had a diagnostic ultrasound only (Table 4). Ten of the 11 cancers were detected by mammogram (Sn = 91 %, Sp = 51 %) and one undetected, a 1-cm size invasive ductal carcinoma. The iBE detected the cancer the mammography missed; conversely, mammography identified the cancers the iBE missed.

**Table 4:** Mammogram accuracy for malignancy detection

	No cancer	Cancer	Total
Mammogram (+)	20	10	32
Mammogram (−)	21	1	21
Total	41	11	52
Measure	Sn = 91 %	Sp = 51 %	

Tables 3 and 4 display the accuracy of malignancy detection of the iBE evaluation and mammography. Table 4 shows the diagnostic mammogram with a total of 32 positive findings. Positive findings for mammograms were defined by the radiologist assigned BIRADS 0, 4, 5, and 6, and negative evaluations were defined as BIRADS 1, 2, and 3. BIRADS 3 is added to the negative evaluation, because BIRADS 3 is considered nonmalignant category. All but one of the BIRADS 0 was eventually determined to be a BIRADS 4 by ultrasound or biopsy. The positive predictive value, negative predictive value, and the accuracy of the mammogram to detect cancer were determined to be 33.3, 95.5, and 59.6 %, respectively. The positive predictive value, negative predictive value, and the accuracy of the iBE to detect cancer were determined to be 10.6, 99, and 74.9 %, respectively.

## Discussion

The iBE demonstrated acceptable sensitivity and specificity in this cohort of patients requiring diagnostic imaging for palpable lesions or abnormal screening mammograms. This study was designed to validate the iBE’s ability to detect lesions in patients with a positive breast exam or imaging.

Mammography and ultrasonography demonstrate sensitivity rates of 85–88 % [4–7] and 93–97 % [7–10] for a general screening and diagnostic population, while the iBE in this population of known detectable findings demonstrated a comparable sensitivity of 85.7 %. The performance of this device meets the threshold of a pre-screening device when compared to the current pre-screening tool, CBE, which has a sensitivity of 50–60 % [11, 12].

The iBE detected all but two of the malignancies, both of which were less than a 1 cm in size. This threshold is an important one for a pre-screening device in low-resource settings. The goal of early detection is to identify malignancies where intervention will improve survival, possibly with reduced need for expensive and morbid adjuvant therapy. Identifying cancers in stages I and II before they become stages III or IV definitely fulfills this criterion in a low-resource environment [13–16]. The iBE is designed to be a pre-screener in order to help identify masses that need to be investigated further; therefore, this amplified sensitivity could be valued to detect potentially significant underlying masses.

Directly comparing the sensitivity of cancer detection demonstrates that both the iBE (83 %) and mammography (91 %) are reliable tools to identify patients with cancer (Tables 3 and 4). While the sensitivity of cancer detection is lower, the specificity of the iBE (74 %) surpasses that of mammography (51 %) indicating that the iBE is superior to mammography at not falsely identifying patients with cancer suggesting that the iBE as a pre-screening tool will be less likely to cause unnecessary stress and anxiety.

Reexamining the iBE results to reflect a patient outcome instead of a single breast or a portion of the breast, we determined that 61 of the 78 patients required a mammogram or an ultrasound for further evaluation of the screening results. Of those 61 patients, 56 (91.8 %) were properly recommended by the iBE for a mammogram or ultrasound; however, 5 (8.2 %) were missed by the iBE that needed a mammogram or ultrasound for further information. Based on the imaging results, 17 patients did not require a mammogram or ultrasound, 4 (23.5 %) of them were identified correctly by the iBE while 13 (76.5 %) were not.

All 78 patients were eligible to participate in this study due to positive imaging, clinical breast exam, or a self-breast exam identifying a suspicious area directing them down the diagnostic pathway, resulting in a 50 % biopsy rate for this study population. This incidental high biopsy rate was beneficial because the biopsy results confirmed the identity/classification of the suspicious masses detected by the iBE or imaging. We demonstrated the iBE’s ability to screen similar to mammography in this cohort. The iBE screenings in this study did not lead to an increase rate of biopsies as the iBE results did not influence the patient’s diagnostic pathway. The high biopsy rate in this study was a result of examining a population on the diagnostic testing pathway.

The iBE is a sensitive tool that is ready for validation in a large screening cohort. This screening study is already underway, and we hope to have a reliable specificity value from that trial before deploying this device in low-resource settings around the world. Because the device is portable, radiation free, and painless and does not require a radiologist, the iBE is an excellent pre-screening device. When used to select patients for further diagnostic workup with mammogram, ultrasound, and/or biopsy, the iBE has the potential to bring access to breast cancer screening and diagnosis to women who currently have little or no access to screening mammography worldwide.

We see the iBE as a potential powerful screening tool for use in the third world countries with limited resources where mammography and clinical breast exam by a trained physician is not readily available. Positive findings by the iBE could then allow for patients to be referred to larger medical centers where ultrasound and mammography may be available and, more importantly, where surgical excision could be performed. Training lay people on the use of the device could potentially provide screening to rural and underserved areas of undeveloped nations where women historically have had no access to breast cancer screening. The iBE device is currently being piloted in a rural area in India where due to limited economic resources, women have essentially no access to mammography and present with late-stage breast cancers. As described in the literature, late-stage breast cancers have poorer prognosis than early stage breast cancers [16]. Due to the significantly lower cost of the iBE compared to conventional mammography, we see this device as a very useful adjunct to breast cancer detection worldwide in underdeveloped, third world nations with limited medical economic resources and a need for improved breast cancer screening. Indeed, a group in Bangalore, India, has recently reported on the use of the iBE in a population of women presenting for annual health exams [17]. Abnormal iBE studies were followed by directed ultrasound or mammography as confirmatory studies. In that population, a sensitivity of 84 % and specificity of 94 % for the detection of image detected breast lesions.

## Conclusions

This validation study demonstrated excellent sensitivity of iBE for the identification of clinically significant lesions in patients presenting for diagnostic imaging. The iBE demonstrates significant potential as a low-cost screening tool in low-resource environments.

## Abbreviations

Acc: Accuracy
BIRADS: Breast Imaging-Reporting and Data System
CBE: Clinical breast exam
CTSRMC: Clinical Trials Scientific Review Committee
DCIS: Ductal carcinoma in situ
iBE: iBreastExam
IRB: Institutional Review Board
NPV: Negative predictive value
PEF: Piezoelectric finger
PPV: Positive predictive value
